# 

**Published:** 1922-02

**Authors:** 


### OUR PRIZE LEAFLETS.

OUR January competition, as we expected, having regard to the universal interest of the subject set, produced several very good efforts. We have decided to divide the prize of Five Guineas, and to award Two Guineas each to *Marcus* and *Persiflage,* whose leaflets are of equal merit, setting out all the necessary facts and exhortations very simply and clearly. One Guinea goes to *Spero,* who is commendably terse and makes effective use of the analogy between the human body and the engine, but would have done better to avoid the words " protein " and " carbohydrates." People more instructed than the school children, for whom our leaflets are intended, are frequently convinced with difficulty that these substances cannot be bought in packets from the chemist.

In this competition *Hygeia* was a good fourth. We like his points about fruit and sweets (" Sweets make for pasty faces and pimples ; fruit makes for clear skins and rosy cheeks ") and on laughter as a digestive. We hope he will try again. Honourable mention is also due to *Tefs, Odd Number,* and *Daddy's Daughter.*

Several competitors tried to pack too many general rules of health into the narrow compass of 300 words. Some concentrated on the art of chewing and ignored the question of what to chew. " Think what your doctors have to put up with," says *Mezzo-Soprano,* reproaching those who grumble if they cannot have their food just when they want it, and forgetting that in a leaflet addressed in part to elementary school children, one must not even seem to countenance irregular mealtimes. Others have not had their audience sufficiently in mind. One, besides lugging in vitamins A, B and C, recommends " a little wine at dinner." Another assumes that the two-course midday dinner is necessarily plainer and wholesomer than the later meal of several courses—a choice hardly within the control of the school child in any case.

---

( 1 )

### Eating and Health.

To be strong and healthy you need food—good, plain, clean food, but you also need to know *what to eat, when to eat* and *how to eat.* If you do not treat food well it will not treat you well, and you may become weak and ill.

WHAT TO EAT.

Foods that will build your body and help you to grow :—Meat, fish, eggs, cheese, milk, peas and beans.

Foods that will keep your body warm and give you strength and power to work :—Butter, dripping, potatoes, rice, sugar and bread.

Foods that will purify your body and keep your blood in good condition :—Vegetables, especially green vegetables, and fruits.

WHEN TO EAT.

It is important that you should eat at regular intervals, and it is equally important that you should not eat cakes, sweets, &c., between those intervals. After you have had a meal the stomach and other organs in your body have to work. It is their business to get all the value they can out of the food you have eaten, but they cannot do this work properly unless you allow them enough time in which to do it.

HOW TO EAT.

The golden rules to remember are :—

DON'T BOLT YOUR FOOD.—If you do, your food will do you more harm than good, and sooner or later you will suffer from indigestion and constipation.

DON'T OVER-EAT.—First, because it is the food digested, not the food swallowed, that counts. Secondly, because it is greedy.

CHEW YOUR FOOD WELL.—To do so is good exercise for your teeth and jaws ; it promotes a plentiful flow of saliva, which mixes with the food and helps it to digest, and it saves your digestive organs a great deal of hard work.

*Marcus.*

---

( 2 )

### Eating and Health.

What you are and what you will become depends very much on what food you eat, and how and when you eat it. An engine needs to be fed constantly with good fuel ; if it is not, it will not give out energy. Nor can *you* give out energy unless you have

PLENTY OF FOOD

at regular times. But it must be the right food. Different sorts of food give your body the different things it needs to keep it well and vigorous, and to make it grow strong. Therefore, eat everything good that is set before you, and

DON'T BE FADDY.

There are three kinds of food—(i.) *body-building* (meat, fish, milk, cheese, eggs, peas, beans, lentils, &c.) ; (ii.) *energy and warmth-giving foods* (butter, margarine, dripping, cream, potatoes, rice, tapioca, &c.) ; and (iii.) *purifying foods* (vegetables, fruits, salads, &c.). You need all these three kinds of food every day ; and don't forget that

SUET PUDDINGS ARE EXCELLENT.

In many kinds of foods there are certain important substances, without which we should not keep well. But the foods containing them must be

FRESH FROM THE LAND.

The more recently your fruit, salads, milk, butter and eggs have been collected from the garden and farm the more good will they do you.

To get the best value out of your food, you must eat it properly. Talk whilst at meals, eat slowly, and always

CHEW YOUR FOOD WELL.

On *no* account get into the habit of eating at odd times between meals. If you do, you will in time spoil your digestion and become ill.

Finally, never be greedy. Get up from the table feeling satisfied, but not " full." Don't rush about directly after your dinner.

*Persiflage.*

---

[*Owing to lack of space, we are compelled to hold over " Spero's " leaflet until next month.*—ED.]

### A Leaflet Competition

*We offer* **THREE GUINEAS** *for the best new and original leaflet of not more than* 300 *words on*

**"THE DANGERS of ALCOHOL"**

The Leaflet must be suitable for circulation in the Public Elementary Schools, and among the Scholars of the Secondary Schools. The result of this Competition will be announced and the successful Leaflet will be printed in the March Number of "The Hospital and Health Review." We reserve the right to republish any of the Leaflets submitted to us.

RULES AND REGULATIONS.

1. This Competition is open to all.
2. Leaflets must be written or typed on unprinted paper on one side of the page only.
3. Competitors must employ a pseudonym.
4. Leaflets must be received by the Editor at "The Hospital and Health Review," 28 Southampton Street, Strand, W.C.2, by **first post on February 16, 1922.** Envelopes must be marked in the top left-hand corner with the word "Leaflet."
5. The Editor's decision is final.

### RECENT APPOINTMENTS.

### Public Health.

Mr. F. D. Ross-Keyt, M.B., Ch.B., D.P.H., to be assistant school medical officer to the North Riding of Yorkshire Education Authority.

Mr. S. Rose, M.B., B.S. (Lond.), L.R.C.P., to be third assistant medical officer for Fulham.

Mr. John Watson Simpson to be assistant medical officer of health for Warwickshire.

Miss Elsie M. Stansfeld, M.B., M.R.C.S., L.R.C.P., to be assistant school doctor to the Brighton Education Authority.

Miss Winifred Kindness, M.B., Ch.B., D.P.H. (Aberdeen), to be assistant school medical officer to the Cornwall Education Authority.

Dr. W. C. Fowler, M.D., B.S. (Lond.), to be medical superintendent at Pinewood, Wokingham, Berks. (Metropolitan Asylums Board).

Mr. Alexander Johnstone, M.A., M.B. (Aber.), D.P.H., to be assistant medical officer and tuberculosis officer for the County of Dumbarton.

Miss Janet Grant, M.B., Ch.B., D.P.H., to be assistant medical officer to the Cardiff Health and Education Committee.

Dr. J. A. Watt to be chief assistant medical officer of health for Derbyshire.

Dr. Victor Farrar Soothill to be Assistant Medical Officer of Health for Ilford, Essex.

Dr. William S. King, M.B., Ch.B. (St. Andrews), to be Medical Officer of Health for Stirling.

Dr. Alexander Johnston, D.P.H., to be Medical Officer of Health for the County of Dumbarton.

### LECTURES ON INFANT AND CHILD WELFARE.

Dr. Eric Pritchard (physician in charge of the Infant Welfare Department) will give a course of twelve lectures on the management and feeding of infants and young children to qualified practitioners, commencing Wednesday, February 8, 1922, at the St. Marylebone General Dispensary, at 6 o'clock in the evening. Students taking this course will be entitled to attend the infant consultations held by Dr. Eric Pritchard on Tuesdays at 11 a.m., and Thursdays at 3 p.m., at the Dispensary ; on these days practical demonstrations will be given as far as opportunity allows on the subjects referred to in the lectures. Opportunities will also be afforded to students of visiting the Nursery Training School, 1, Wellgarth Road, Golder's Green (5 minutes from the Tube Station), and of seeing the same methods employed there in dealing with resident infants. The visits will be paid on Saturday afternoons. The fee for the course is two guineas. For further information, tickets, &c., apply to the secretary, 77 Welbeck Street, Cavendish Square, W. 1.

A course of elementary lectures on infant care, especially intended for crèche nurses and probationers, is being given by Dr. Flora Shepherd, Medical Officer to the Hornsey and Surrey Square Infant Welfare Centres, at Carnegie House, 117, Piccadilly, W. 1. The lectures, which began on January 26, are to be given on Thursdays, from 7.30 to 8.30, until April 20. An advanced course will be held on Mondays, from 6.0 to 7.0, from January 23 until April 10. Tickets can be obtained from Miss Halford, hon. secretary, National Association for the Prevention of Infant Mortality, 117, Piccadilly, W. 1.

During 1921 the sum collected by the Metropolitan Hospital Sunday Fund amounted to £108,880, being £12,178 less than the previous year's total. Collections in various places of worship realised £50,267—a decrease of over £3,000, while donations amounted to £22,311, or £8,695 less than in 1920. The working expenses for 1921 were £4,730, as against £3,828. Hospital Sunday this year is to be held on June 18, and as 1922 is the Fund's jubilee year, it is hoped that a record collection will be made.

